# Supplementary material for: Feasibility and acceptability of nationwide HPV vaccine introduction in Senegal: Findings from community-level cross-sectional surveys, 2020
Source: PLOS Glob Public Health. 2022 Apr 6;2(4):e0000130. doi: 10.1371/journal.pgph.0000130 (PMC10021483; doi:10.1371/journal.pgph.0000130)
Supplement: Attachment — Submitted filename: HPV PLos GH Response.docx [file pgph.0000130.s001.docx]

**Response to Reviews**

PGPH-D-21-00369
Feasibility and acceptability of nationwide HPV vaccine introduction in Senegal: findings from community-level cross-sectional surveys, 2020
PLOS Global Public Health

Dear Dr. Doshi,

Thank you for submitting your manuscript to PLOS Global Public Health. After careful consideration, we feel that it has merit but does not fully meet PLOS Global Public Health’s publication criteria as it currently stands. Therefore, we invite you to submit a revised version of the manuscript that addresses the points raised during the review process.

Thanks for submitting a good manuscript on a relevant topic. There are some minor comments to be addressed before a final decision can be reached.

Please submit your revised manuscript by Nov 26 2021 11:59PM. If you will need more time than this to complete your revisions, please reply to this message or contact the journal office at [globalpubhealth@plos.org](mailto:globalpubhealth@plos.org). When you're ready to submit your revision, log on to <https://www.editorialmanager.com/pgph/> and select the 'Submissions Needing Revision' folder to locate your manuscript file.

Please include the following items when submitting your revised manuscript:

- A rebuttal letter that responds to each point raised by the editor and reviewer(s). You should upload this letter as a separate file labeled 'Response to Reviewers'.
- A marked-up copy of your manuscript that highlights changes made to the original version. You should upload this as a separate file labeled 'Revised Manuscript with Track Changes'.
- An unmarked version of your revised paper without tracked changes. You should upload this as a separate file labeled 'Manuscript'.

Guidelines for resubmitting your figure files are available below the reviewer comments at the end of this letter.

We look forward to receiving your revised manuscript.

Kind regards,

Charles Anawo Ameh
Academic Editor
PLOS Global Public Health

Journal Requirements:

1. We note that participants provided oral consent. Please state in the Methods:
   - Why written consent could not be obtained
   - How oral consent was documented

We have revised this.

For more information, please see our guidelines for human subjects research: <https://journals.plos.org/plosone/s/submission-guidelines#loc-human-subjects-research>

2. You indicated that ethical approval was not necessary for your study. We understand that the framework for ethical oversight requirements for studies of this type may differ depending on the setting and we would appreciate some further clarification regarding your research. Could you please provide further details on why your study is exempt from the need for approval and confirmation from your institutional review board or research ethics committee (e.g., in the form of a letter or email correspondence) that ethics review was not necessary for this study? Please include a copy of the correspondence as an ""Other"" file.

The Senegal Ministry of Health considered this to be a program evaluation and did not require ethical approval. This was an internal country decision. I have attached some additional documentation in the other file from the CDC side.

3. Please provide separate figure files in .tif or .eps format only, and remove any figures embedded in your manuscript file.  If you are using LaTeX, you do not need to remove embedded figures.

For more information about figure files please see our guidelines:

<https://journals.plos.org/globalpublichealth/s/figures>

4. We do not publish any copyright or trademark symbols that usually accompany proprietary names, eg (R), (C), or TM  (e.g. next to drug or reagent names). Therefore please remove all instances of trademark/copyright symbols throughout the text, including Microsoft Excel® on page 6.

 Done

5. Please update the completed 'Competing Interests' statement, including any COIs declared by your co-authors. If you have no competing interests to declare, please state "The authors have declared that no competing interests exist". Otherwise please declare all competing interests beginning with the statement "I have read the journal's policy and the authors of this manuscript have the following competing interests:"

 Done

6. Since your data is not available for proprietary reasons, please explain via email why the data is not available. Please also include the contact information for the third party organization that should be contacted should other researchers want to request access to this data and please include the full citation of where the data can be found. We also request that you verify with us via email that any researcher will be able to obtain the data set in the same manner that the you have obtained it. If you feel you are unwilling or unable to adhere to this policy, please explain your reasons by return email and your exemption request will be escalated to the editor for approval. Your exemption request will be handled independently and will not hold up the peer review process, but will need to be resolved should your manuscript be accepted for publication. One of the Editorial team will be in touch if they require more information.

 The data is the property of the Senegal Ministry of Health and Social Action (MoHSA). Only they can provide access to the data. They must be contracted directly for data access.

7. Please amend your detailed Financial Disclosure statement. This is published with the article, therefore should be completed in full sentences and contain the exact wording you wish to be published.

i). State the initials, alongside each funding source, of each author to receive each grant.

ii). State what role the funders took in the study. If the funders had no role in your study, please state: “The funders had no role in study design, data collection and analysis, decision to publish, or preparation of the manuscript.”

Revised.

Please review your reference list to ensure that it is complete and correct. If you have cited papers that have been retracted, please include the rationale for doing so in the manuscript text, or remove these references and replace them with relevant current references. Any changes to the reference list should be mentioned in the rebuttal letter that accompanies your revised manuscript. If you need to cite a retracted article, indicate the article’s retracted status in the References list and also include a citation and full reference for the retraction notice.

Reviewer #1: Thank you for your interesting article on a very important topic.
The data presented is extensive and provides useful information that can inform ongoing implementation efforts for the national HPV vaccination program in Senegal.

There are a few issues that I suggest need to be addressed in order to strengthen the article (please see the attached document).

**Abstract**

It would be helpful to include a summary of the main research question in the ‘Background’ section, i.e. what was the aim of this study, and how does it address a research gap?

This is a style preference and the first sentence of methods describes the overall aim of the study. We could move it to background if reviewers and the journal prefers, but we prefer it in the background.

Line 46: please specify the number of community leaders

Revised

Line 48: please check data reported (should this not be “52.5 – **94.8**%”)?

Revised

**Methods**

Line 103: suggest changing the word ‘interviews’ to ‘structured surveys’ as this is more accurate. Suggest replacing the word ‘interviewed’ with ‘surveyed’ throughout your methods section.

Revised

Please describe how the study questionnaire was developed, e.g. was it adapted from validated questionnaires? Who was it piloted with? What kinds of adaptations were made?

This section has been revised to include additional details

Please describe administration of the survey in more detail. Was it administered by research staff for all participants or was it self-administered in some cases?

The surveys were conducted by trained data collectors that were selected by the MoH, EPI, WHO. We have added more details.

In you ‘data collection’ section, please describe how the data was handled to ensure privacy e.g. was the data only available to trained researchers?

Revised to include more details, please note as stated names were never collected and data collectors did not have access to the server. Once uploaded, the data was no longer accessible on the tablet.

**Results**

Please include the number that is relevant to the reported percentage for all results. For example, line 164, “49.4% were female” should read “n=38 (49.4%) were female”

While we do not disagree with the reviewer that is another correct way to present the data, we looked at other cross-sectional surveys in the journal, and results were reported similarly to ours, so we have left the results as is.

Lines 179-180: it is best to report the percentages directly after the relevant stakeholder, i.e. “Only a small proportion of parents (n=43, 28.3%) and community leaders (n=27, 33.8%) knew that…..”

Thank you. We have revised this.

**Tables**

Table 2: please review the subheading *“If an eligible girl is absent the day of vaccination*”* and consider rewording this to *“what would you do if an eligible girl is absent the day of vaccination”*

Thank you. We have revised this.

Table 2: please review the subheading *“how do you determine the eligible population for the HPV vaccine dose 2?”* This seems to be repeated. Should the first of these refer to the first dose? Please check and amend appropriately

Thank you. We have revised this.

**Formatting**

Please ensure consistency in use of the acronym ‘HCW’ – in some instances, e.g. line 41, you use ‘HCWs’ while in other instances, e.g. line 45, you use ‘HCW’.

Thank you. We have reviewed and revised

Similarly, please ensure consistency in your use of the acronym ‘cHCW’ – in some instances, this is used while in other instances, e.g. line 208, it is not used.

Thank you. We have reviewed and revised.

Line 141: please use consistency in formatting of numbers (i.e. this should read “five supervisors and **eleven** interviewers”…

This is not meant to be inconsistent. My general rule of thumb is do not start a sentence with a number so the number must be written out (e.g. Five). Additionally, I generally only write out numbers if then are 1-10. Depending on the journal preference, I am happy to do either.

Reviewer #2: This is a clearly written paper reflecting a well done study on an important topic for the global immunization and cancer control communities. Cervical cancer, the number one or two cancer killer of women in much of the developing world is highly preventable with safe and very effective vaccines when given to girls before the onset of sexual activity. GAVI’s ability to procure affordable HPV vaccines and supply them to the poorest countries represents great progress in global cancer control, and the success of the pilot and early national program in Senegal is very encouraging and important.

This study of knowledge and practices of HPV immunization in randomly selected communities in Senegal is well conceived, rigorously conducted, properly analyzed, and intelligently discussed. The discussion also provides useful advice for immunization programs in future GAVI countries and beyond. I have only a few comments and suggestions.

I think the introduction could be strengthened with some additional information such as Senegal’s immunization coverage with measles and DTP vaccines; data, if available yet, on National HPV coverage from WHO/UNICEF data or national surveys; and UNICEF estimates on the proportion of nine year old girls in school. The survey asks HCW and school personnel to estimates how many 9 year olds attend school, but they don’t know. Perhaps outside data may help to clarify this.

Thank you for the suggestion. I thought a lot about this and do not think in strengthens the overall introduction for a few reasons. The first these are childhood vaccinations and now we are talking about a new age group. Additionally, the introduction is very focused on HPV and not so much on immunization as whole in Senegal, I could not find a paragraph in the introduction where the addition of these data flowed naturally.

I was initially unclear what was meant by “community” delivery of the vaccine. Is outreach to girls who don’t attend school or who missed a dose at school all of it, or are there other mechanisms of community delivery?

There were specific community posts that offered vaccination. This is clarified in the texts.

It is mentioned in the methods section and discussion that although the communities studied are randomly chosen, from that point on this is a convenience sample. The interviewee school staff and parents were chosen by a local HCW who would likely choose a school and individuals he or she knew would understand the program well. Why was this method of selection chosen? The schools and parents could have been randomly chosen. A brief discussion of this would be useful, and mentioning that this is a convenience sample in the abstract might also be appropriate.

Thanks for the comment. This was mainly for ease of finding a subject. Even if randomly chosen, it would not have been a random sample as it would have been very challenging to create a complete list of all parents in the community.

Additionally, it was already stated that is a convenience sample in the abstract. “During February to March 2020, we conducted surveys to assess the awareness, feasibility, and acceptability of the HPV vaccination program with a cross-sectional convenience sample of healthcare workers (HCWs), school personnel, community healthcare workers (cHCWs), parents, and community leaders from 77 rural and urban health facility catchment areas.”

Overall, an important and well done study.

Thank you for the helpful comments.
